# Use of subcutaneous immunoglobulin in stiff person syndrome: Case series

**DOI:** 10.1097/MD.0000000000025260

**Published:** 2021-03-26

**Authors:** Salman Aljarallah, Scott D. Newsome

**Affiliations:** a King Saud University, College of Medicine, Riyadh, Saudi Arabia; b Johns Hopkins Hospital, Stiff Person Syndrome Center, Baltimore, MD, USA.

**Keywords:** autoimmune diseases, case reports, immunoglobulin, stiff person syndrome

## Abstract

**Introduction:**

Intravenous immunoglobulin (IVIG) has been shown to be effective for the treatment of stiff person syndrome (SPS). However, some patients might not tolerate it. We report the tolerability profile of subcutaneous immunoglobulin (SCIg) in patients with SPS who did not tolerate IVIG. To our knowledge, the use of SCIg in SPS has not been reported before in a case series.

**Patient concerns:**

The five patients included in this case series presented with various combinations of symptoms of spasms, axial and limb stiffness, and exaggerated responses to outside stimuli. These symptoms often lead to gait and functional impairment.

**Diagnosis:**

Patients were diagnosed with classic SPS as they met the clinical criteria, which require the presence of spasms, axial rigidity, and hyperexcitability.

**Interventions:**

Subcutaneous immunoglobulin infusion.

**Outcomes:**

Five patients were identified that were treated with SCIg. Three tested positive for serum anti-glutamic acid decarboxylase 65 antibodies prior to any treatment. The mean age at SCIg initiation was 33 years (range: 22–47). The mean duration of SPS prior to SCIg initiation was 5.9 years (range: 2.5–7). All patients used IVIG for at least two months (up to 18 months) but switched to SCIg due to IVIG side effects. Duration of SCIg use ranged from 4 months to 6 years (mean, 19.2 months). Upon switching to SCIg, the SPS symptoms remained stable. SCIg was well-tolerated in most as only one patient discontinued SCIg due to side effects.

**Conclusion:**

This case series highlights that SCIg could be a treatment option for patients with SPS, especially when IVIG is not feasible. Injection site reactions might be a limiting factor in some patients treated with SCIg. Prospective controlled studies are needed to confirm SCIg treatment durability and efficacy.

## 1 Introduction

Stiff person syndrome (SPS) is a rare immune-mediated disorder characterized by disabling muscle spasms, axial rigidity, and hyperexcitability. Most patients have antibodies to glutamic acid decarboxylase 65 (GAD65) enzyme, which has an unclear role in the disease pathogenesis.^[1]^ In some studies, up to 20% of patients with SPS will not have these autoantibodies.^[2]^ Intravenous immunoglobulin (IVIG) was shown to help SPS in a placebo-controlled randomized clinical trial.^[3]^ However, challenges associated with IVIG exist including poor tolerability, monthly infusion visits, need for IV access, and side effects related to the large volume of protein administered (aseptic meningitis, renal complications, and/or increase thrombosis risk). Hence, subcutaneous delivery of immunoglobulin (SCIg) has emerged as an alternative with comparable efficacy in other conditions such as inflammatory polyneuropathies.^[4]^ To our knowledge, the use of SCIg in SPS has not been reported before in a case series.

## 2 Materials and methods

This is a case series of patients with SPS treated at the Johns Hopkins Stiff Person Syndrome Center. The study population included any patient with the diagnosis of SPS, who was treated with SCIg from 1997 to 2019. All patients included needed to fulfill the modified SPS clinical diagnostic criteria which require the presence of the following; insidious onset of stiffness/rigidity in the limbs and/or axial musculature, co-contraction of agonist and antagonist muscles (per history, examination, or by electromyography [EMG]), superimposed spasms that are triggered by unexpected stimuli or startle, response to benzodiazepines, and absence of any other neurologic disease that can cause spasms or rigidity.^[5]^ Patients were excluded if there was another cause for their neurological/musculoskeletal signs and symptoms. All patients underwent magnetic resonance imaging (MRI) of the brain and whole spine to exclude any disorder that can cause spasms, stiffness, spasticity, or rigidity. All patients underwent nerve conduction studies and EMG on the affected limb to exclude a peripheral neuromuscular disorder.

Serum anti-GAD65 antibodies were tested using various assays in different commercial labs. The assay obtained depended on what was available at the time of the test request and patient's insurance preference. A patient is considered “seronegative” if anti-GAD65, antiamphiphysin, and glycine receptor antibodies were not detected in the serum.

Given the retrospective nature of this study and varying number of clinic visits amongst the patients, the exact frequency and severity of patient's spasms were not captured. The modified Rankin Score (mRS) was used to describe the degree of disability/dependency. All patients were assessed by a neurologist (senior author) with expertise in SPS.

### 2.1 Standard protocol approvals, registrations, and patient consents

Data was collected and submitted for publication in compliance with institutional ethical standards after obtaining approval from the Institutional Review Board at Johns Hopkins School of Medicine (IRB00154798). All patients (or guardians) signed consents allowing data collection and publication.

## 3 Results

### 3.1 Patient 1

A 31-year-old man with Sjogren's syndrome developed painful foot and leg spasms. The spasms would rapidly spread to involve his arms, back, and torso leading to falls. Loud and abrupt high-pitched noises would trigger the spasms. Examination showed axial stiffness and spasms of the thoracolumbar paraspinal and hip musculature. EMG demonstrated co-contraction of tibialis anterior and gastrocnemius muscles. Baseline and follow up serum anti-GAD65 antibodies ranged from 2.3 U/mL to 288 U/mL (normal < = 1.0). Oral diazepam resulted in symptom relief, but higher doses were intolerable, prompting IVIG treatment (0.4 g/kg per day for 5 days each month). The frequency and severity of muscle spasms and stiffness improved with IVIG (see Table 1). However, during and after IVIG, he experienced severe headaches, myalgias, nausea, vomiting, and fatigue. This resolved over weeks but re-occurred with each IVIG course despite premedication with methylprednisolone, acetaminophen, diphenhydramine, and fluids. He subsequently transitioned to weekly SCIg (0.5 g/kg/week). Evaluation at 12 and 14 months after initiation revealed acceptable SPS symptom control with a reduction in spasm frequency and intensity. His mRS score improved from 2 to 1, and walking speed measured by a timed 25-foot walk test also improved. He stopped taking diazepam and only requires a low dose of clonazepam. He reported mild injection site reactions with SCIg which he tolerates

**Table 1:** Summary and description of patients and their response to subcutaneous immunoglobulin.

	Age (at SCIg initiation), gender	anti-GAD65 antibody	SPS symptoms	Body regions affected	Duration of SPS prior to SCIg	Duration of IVIG treatment	Reason for IVIG discontinuation	Total IVIG dose (monthly)	Latest SCIg total dose (monthly)	Duration of SCIg treatment	SCIg side effects
1	33 yr/o, Male	Present	Spasms, stiffness, increased startle	Feet, legs, arm, torso, entire body	2.5 yr	2 mo	Severe headache, myalgias, nausea, vomiting, fatigue	2 g/kg	2 g/kg	18 mo	Injection site reactions
2	47 yr/o, Female	Absent	Axial and limb stiffness, spasms, agoraphobia	Torso, back, neck, arms, legs	3 yr	3 mo	Hypertension, aseptic meningitis	2 g/kg	0.4 g/kg	75 mo	None
3	36 yr/o, Female	Present	Spasms, stiffness, exaggerated startle, myoclonus, dysphagia, anxiety, agoraphobia	Legs, arms, abdominal muscles, neck, back	7 yr	18 mo	Wearing off, unable to tolerate more frequent doses, fatigue, skin lesions/hives	2 g/kg	1.6 g/kg	12 mo	None
4	22 yr/o, Female	Present	Spasms, limb stiffness, hyperexcitability	Feet, legs, back, arms	6 yr	3 mo	Allergic skin reaction despite premedication and very slow infusion rate, major spasm crisis following third dose	2g/kg	1 g/kg	8 mo	None
5	25 yr/o, Female	Absent	Spasms, stiffness, exaggerated startle, anxiety	Arms, legs, torso, back	6 yr	4 mo	Bronchospasm and breathing difficulties	2 g/kg	1 g/kg	4 mo	Breathing difficulties and persistent SPS symptoms

anti-GAD65 = anti-glutamic acid decarboxylase 65, g = grams, IVIG = intravenous immunoglobulin, kg = kilograms, SCIg = subcutaneous immunoglobulin, SPS = stiff person syndrome, yr/o = years old.

### 3.2 Patient 2

A 53-year-old female noted difficulty walking and running at the age of 32 years. Twelve years later, she noted insidious onset of back discomfort and tightening/stiffness of her torso/chest/abdominal musculature, which eventually involved her extremities. She also experienced painful, episodic muscle spasms that would start abruptly, occur on any of the body areas mentioned, and typically last for several minutes at a time. Anxiety, stress, cold temperatures, and being in open spaces provoke her symptoms. Examination showed torso stiffness and spasms of the thoracolumbar paraspinal muscles and both trapezii. Serum anti-GAD65 antibodies were negative. The EMG was normal showing no signs of denervation, continuous muscle fiber activity, or cocontraction of agonists and antagonists muscles in the legs. MRI of the brain, cervical, and thoracic spine only showed mild neural foraminal stenosis. Diazepam helped her body pain and stiffness but caused paradoxical insomnia. IVIG therapy (0.4 g/kg per day for 5 days each month) resulted in aseptic meningitis and severe hypertension. She was switched to monthly SCIg (0.4 g/kg), which has been well-tolerated. She reported improvement of her SPS symptoms with SCIg, and her mRS improved from 3 to 2. She preferred staying on a low-dose of SCIg, which was enough to control her symptoms. Insurance-related interruption of SCIg for a couple of months resulted in the recurrence of disabling spasms and stiffness.

### 3.3 Patient 3

A 30-year-old female with lupus and Sjogren's syndrome started experiencing stiffness in her legs and arms with painful spasms along with abdominal muscle spasms. Symptom triggers include unexpected sound or touch. Her walking and use of her extremities were severely limited due to stiffness resulting in wheel-chair dependence. Work-up was remarkable for mild spinal fluid pleocytosis and elevated serum anti-GAD65 antibodies (>250 IU/ml). Plasmapheresis resulted in transient improvement of symptoms. IVIG resulted in remarkable improvement in her arm and torso stiffness (0.4 g/kg per day for 5 days each month). She was able to open her right hand for the first time in 2 years. However, she experienced disabling wearing-off of IVIG requiring more frequent dosing, which caused severe headaches and skin reactions/hives. At this point, she was switched to weekly SCIg (0.4 g/kg/week), which has been well-tolerated and led to the improvement of her functional status from a mRS of 3 to 1. During the course of her SCIg treatment, an insurance-related interruption occurred for a few months resulting in recurrence of disabling spasms, rigidity, and falls.

### 3.4 Patient 4

A 22-year-old female developed back spasms associated with painful flexion of both arms at the age of 15 years. Symptoms progressed over months to involve her feet. Triggers included unexpected noises/startle, stress, and infections. She became wheelchair-dependent and experienced 3 generalized seizures over 3 years. The examination demonstrated thoracolumbar paraspinal spasms, stiffness in her arms and legs, and dystonic-like posturing of her feet. EMG showed continuous muscle fiber activity in the tibialis anterior and gastrocnemius muscles. Serum anti-GAD65 antibodies was 26 nmol/L and CSF anti-GAD65 antibodies were 0.03 nmol/L (normal < 0.02). Diazepam, tizanidine, and dantrolene only partially controlled her spasms. Hence, monthly IVIG (0.4 g/kg per day for 5 days each month) was started leading to improvement in her mobility. However, IVIG was stopped after 6 months due to intolerance (headaches & skin reactions despite corticosteroid and antihistamine premedication). She started SCIg (0.25 g/kg/week), which was better tolerated, and upon follow up several months later, her SPS symptoms improved, and her neurological worsening stabilized but remained functionally limited with a mRS of 4.

### 3.5 Patient 5

A 25-year-old female with asthma developed progressive right leg stiffness/spasms and gait dysfunction. Symptoms progressed to involve her left side and torso, causing a sense of chest tightening interfering with breathing. The main triggers included abrupt loud noises, large crowds, unexpected touch, and certain positions. The examination was notable for stiffness in the right leg and torso, and cervical and thoracolumbar paraspinal muscle spasm. Work-up was unremarkable, including a whole-body fluorodeoxyglucose positron emission tomography scan and MRI of the brain, cervical and thoracic spinal cord. Anti-GAD65, anti-amphiphysin, and glycine receptor antibodies were not detected in the serum. Diazepam, clonazepam, and pregabalin led to transient improvement of her symptoms. Baclofen pump ameliorated her severe spasms, but she developed a tolerance to this therapy.

Six years from symptom onset, she started monthly IVIG (0.4 g/kg per day for 5 days each month) due to ongoing symptoms but developed bronchospasm during her fourth month of treatment that required epinephrine. IVIG re-challenge with extra premedication resulted in further bronchospasm prompting discontinuation of IVIG. Her SPS worsened off IVIG, so she was started on SCIg (0.25 g/kg/week). She reported improvement in her SPS symptoms and walking speed. However, she developed escalating side effects concerning for worsening hypersensitivity reactions (breathing issues) after each SCIg treatment that eventually resulted in discontinuation of SCIg with her fourth month of treatment. Her mRS score stayed at 2.

## 4 Discussion

To our knowledge, this is the first case series reporting the use of SCIg in SPS. All patients experienced typical symptoms and signs consistent with classic SPS phenotype (non-paraneoplastic). The disease burden of these patients worsened over time requiring escalation to an immune based therapy. Most patients in this case series tolerated SCIg with only one patient (who had pre-existing reactive airway disease) discontinuing this treatment due to a suspected evolving hypersensitivity reaction.

IVIG treatments are thought to help immune-mediated disorders by different mechanisms. These include blocking the Fc receptor on the surface of effector phagocytes,^[6]^ suppression of dendritic cells,^[7]^ and binding to circulating pathogenic autoantibodies leading to accelerated antibody elimination or downregulation of production.^[6]^ Also, IVIG has been shown to reduce inflammatory responses by upregulation of regulatory T lymphocytes.^[8]^ These mechanism of actions are likely the same for SCIg since the treatment effects on various autoimmune disorders appears similar. However, there have been no large clinical trials comparing IVIG and SCIg in neuroimmunological disorders. A metanalysis of eight small studies showed no major difference in efficacy, but SCIg was more tolerable.^[9]^ This is in line with our data, where all the patients experienced better tolerability with SCIg while keeping their SPS stable or improved from subjective and objective markers of disease burden (mRS and timed 25-foot walk).

Possible reasons that SCIg is better tolerated than IVIG include slower rate of systemic exposure/absorption of immunoglobulin, different escalating dosing regimens, difference in pharmaceutical properties (e.g., stabilizer, pH, sodium content, osmolality, IgA content), shorter infusion times, etc. Furthermore, the reported improvement of SPS symptoms in some patients after transitioning to SCIg may be attributed to the lower side effect profile compared to IVIG, as pain and stress can exacerbate SPS symptoms. In addition to offering a good tolerability profile, the lack of need for an infusion center and/or need for long-term nursing involvement, SCIg may confer a cost advantage over IVIG, as demonstrated in previously published data.^[10,11]^ While the ability to self-administer immunoglobulins provides another important advantage of SCIg over IVIG, injection site reactions can be a limiting factor for some patients, especially with larger doses and a longer duration of therapy. SCIg also offers an advantage over plasma exchange. Plasma exchange has been reported as a potentially effective treatment for SPS in case reports and small case series, especially in those patients who fail first line therapies.^[12,13]^ However, this treatment is often reserved for acute exacerbations and not for maintenance therapy since it is considered to be more challenging to administer than immunoglobulin therapies. Plasma exchange is most commonly performed via central venous access and typically requires hospitalization for the treatments. Moreover, it can be associated with catheter thrombosis/infections, clinically significant anemia, and hemodynamic changes during treatment.

Limitations of this case series include the small sample size, the observational nature, and the lack of a control arm. Additionally, given the retrospective nature of this study and the varying number of clinic visits amongst the patients, the exact frequency and severity of the patient's spasms/stiffness were not consistently captured. Hence, we are unable to make strong conclusions about the long-term efficacy of SCIg in SPS and whether this is a better treatment option than IVIG beyond differences in tolerability. A randomized, double-blind, head-to-head trial may help determine whether there are major differences between SCIg and IVIG in SPS.

## 5 Conclusion

SCIg may be a reasonable and safe alternative for patients with SPS who do not tolerate IVIG, although allergic and injection site reactions can be a limiting factor in some patients. Controlled studies are needed to confirm SCIg treatment durability and efficacy in SPS.

## Author contributions

**Conceptualization:** Scott Newsome.

**Data curation:** Salman Aljarallah.

**Methodology:** Scott Newsome.

**Supervision:** Scott Newsome.

**Writing – original draft:** Salman Aljarallah.

**Writing – review & editing:** Salman Aljarallah, Scott Newsome.
